# Advance MR evaluation of synchronous multifocal osteosarcoma with pathologic fracture

**DOI:** 10.1259/bjrcr.20210015

**Published:** 2021-04-15

**Authors:** Rosy Setiawati, Eveline Stephanie Lay, Valentina Testini, Paulus Rahardjo, Mouli Edward, Sjahjenny Mustokoweni, Giuseppe Guglielmi

**Affiliations:** 1 Department of Radiology, Faculty of Medicine, Universitas Airlangga, Surabaya, Indonesia; 2 Department of Radiology, Barletta University Campus UNIFG, Barletta, Italy; 3 Department of Orthopedics and Traumatology, Faculty of Medicine, Universitas Airlangga, Surabaya, Indonesia; 4 Department of Pathology, Faculty of Medicine, Universitas Airlangga, Surabaya, Indonesia; 5 Department of Clinical and Experimental Medicine, Foggia University School of Medicine, Viale L. Pinto 1, 71121 Foggia, Italy; 6 Department of Radiology, School of Medicine, Foggia University, Foggia, Italia

## Abstract

**Objective:**

Synchronous multifocal osteosarcoma is a rare condition in which the osteosarcoma presents with multiple bone lesions at the time of diagnosis, usually without any visceral metastases. The first case was described in early 1930s by Silverman. To report a case of synchronous multifocal osteosarcoma in adolescent with pathologic fracture.

**Methods:**

An 18-year-old girl presented with a painful mass in the right thigh of 4 months’ duration and a history of thigh bone fracture a month ago. Patient’s medical records and family history was unremarkable. Physical examination showed angulation and shortening at right femoral region with tenderness and swelling. Initial radiograph and magnetic resonance (MR) images showed multiple lesions in right femoral shaft and pelvic bone with primary tumor in right distal femur with pathologic fracture and multiple bone marrow lesions found in the contralateral bones. Imaging and histopathological results supported the diagnosis of synchronous multifocal osteosarcoma. After following the chemotherapy as the treatment of choice, the radiograph and MRI evaluation were done and showed reduction of the mass size with union of the destructed part with the formation of callus. The advance MRI revealed reduction of the overall mass and the composition of the viable area compared to previous study. The patient had satisfying response to chemotherapy series and a better functional outcome on subsequent visits.

**Results:**

Diagnosis of synchronous multifocal osteosarcoma was based on patient and family history and finding of multiple lesions in the MR images, meanwhile the plain radiograph only revealed the primary tumor. Amstutz described multifocal osteosarcoma as presence of one primary tumor and several smaller lesions. Most recent reviews concluded that multifocal osteosarcoma is bone-to-bone metastatic process rather than multicentric origin. The limitation in this case was absence of thoracic CT which is suggested to rule out any pulmonary metastases instead of routine chest radiograph.

**Conclusion:**

Although satisfying improvement was clinically achieved, further advanced MRI would be indicated to evaluate the progression of tumor and its respond to therapy.

## Introduction

Osteosarcoma is the most common primary malignancy of the bone with tendency for local invasion and early metastases. It occurs most commonly in patients between 5 years of age and early adulthood. The peak of incidence in the elderly has been associated with pre-existing Paget’s disease and prior radiation therapy. It is an osteoid-producing malignancy arising from mesenchymal origins.^1^

Osteosarcoma of the adolescent most often develop at the metaphysis of the lower extremity long bones (~75% of cases), and the relationship between hormonal changes of puberty and/or physiologic bone growth and the pathogenesis of osteosarcoma was suggested.^2^

Multifocal osteosarcoma is defined as the presence of tumor at two or more skeletal sites without any evidence of pulmonary metastases (incidence around 1.5–5.4%).^3^ It may be synchronous, presenting with two or more bone lesions at time of diagnosis, or metachronous, which the new tumors develop after the initial treatment, involving more than one bone.^4^ The first case of multifocal osteosarcoma was described in 1930s by Silverman.^5^

Multifocal osteosarcoma typically presents with one primary large tumor and several smaller bone lesions.^6^ Many literatures stated the prognosis of synchronous multifocal osteosarcoma being poorer compared to the conventional osteosarcoma. Here, we present a case of synchronous multifocal osteosarcoma with complication of pathologic fracture at the main site of the tumor.

## Case report

An 18-year-old girl presented with a painful mass in her right thigh for the past 4 months before admitted to the hospital. The patient also reported a history of thigh bone fracture due to accidentally dropped while being carried by his uncle a month ago. Her medical and family histories were unremarkable.

Physical examination showed angulation and shortening at the femoral region with, tenderness and soft tissue swelling at the anteromedial aspect of the thigh above the right knee

**Figure 1.**
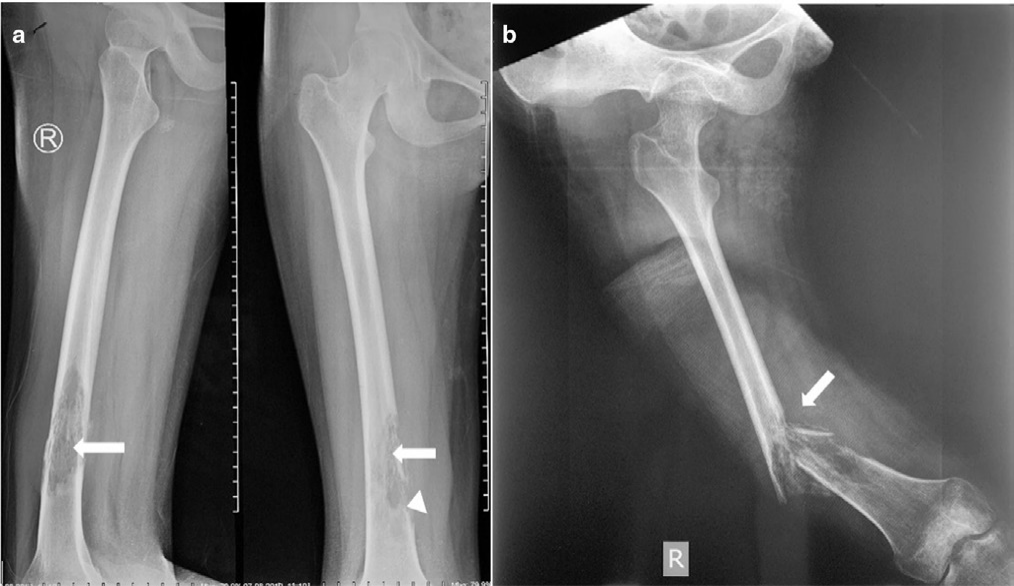
(a) Initial radiograph (07/08/2019) AP and lateral views showed osteolytic lesion (arrow) in the one third distal portion at the anteromedial aspect of femur diaphysis with wide transition zone and periosteal elevation (arrow head). (b) First follow-up images of lateral radiograph (01/11/2019) showed pathologic fracture and bone destruction (arrow) at distal femur diaphysis with a bulky soft tissue mass. AP, anteroposterior.

**Figure 2.**
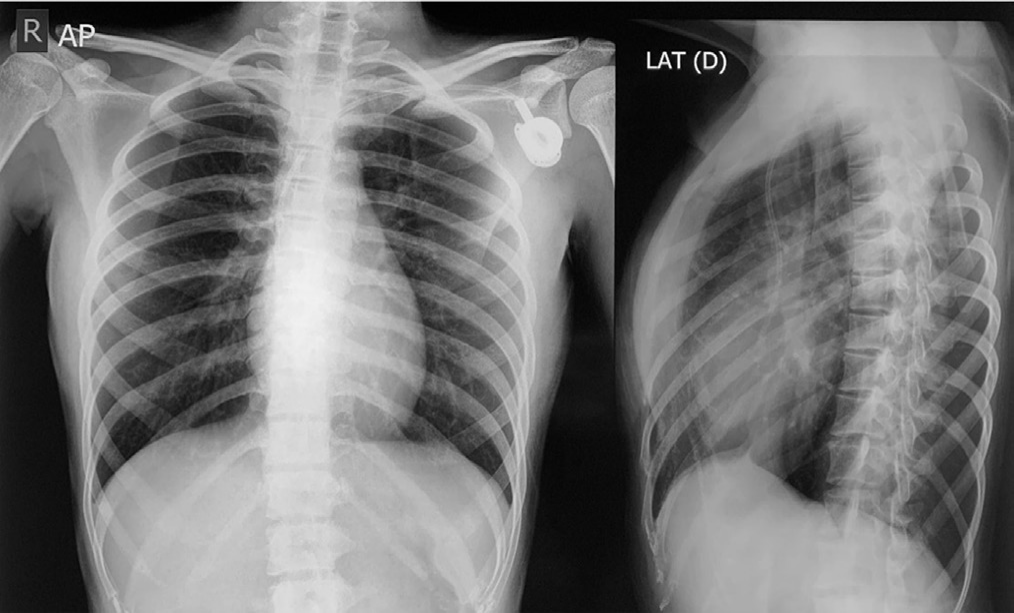
Anteroposterior and lateral chest radiograph showed neither visible nodules nor any metastases lesion.

Initial radiograph was presented, showed osteolytic lesion in one-third distal portion at the anteromedial aspect of right femur diaphysis with wide transition zone and periosteal elevation (Codman triangle) (Figure 1A). Plain radiograph was obtained 3 months later of the right thigh showed a pathologic fracture at the distal femoral shaft and bone destruction in one-third distal portion of the femur diaphysis (Figure 1B). Chest radiograph showed no evidence of pulmonary metastasis(Figure 2).

**Figure 3.**
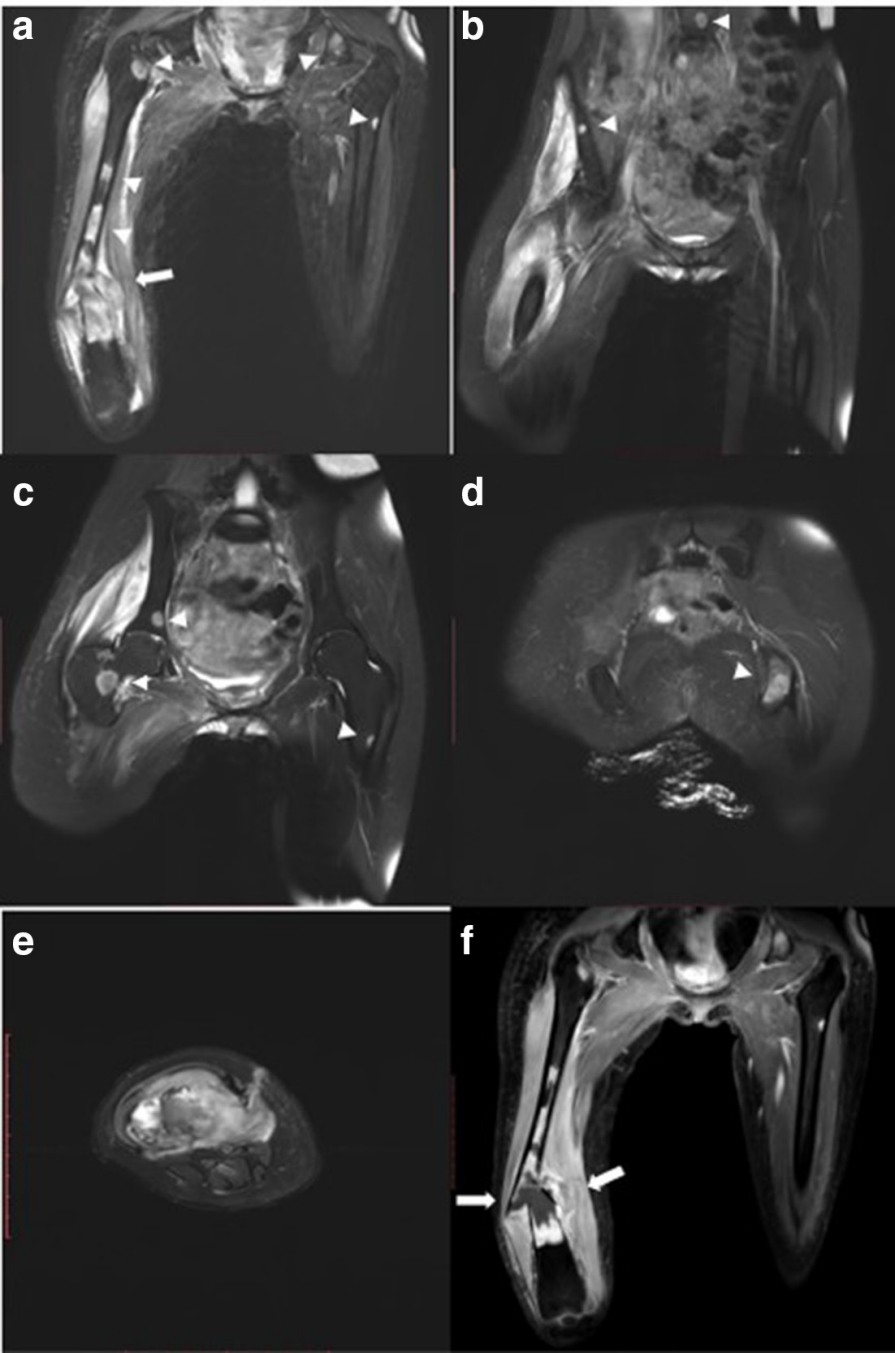
Initial MRI examination (19/11/2019) with T1 Fat Sat MRI images with intravenous gadolinium contrast (a–d) On coronal images showed bone destruction in one-third distal portion of right femur diaphysis to mid-femur shaft with bulky soft tissue mass and pathological fracture in the distal femoral shaft (white arrows). Multiple skip lesions in the fifth lumbar vertebra, right mid-femur shaft, right proximal femur just below the femoral neck, left proximal femur, right iliac wing bone, and left ischium bone (white arrow heads). (e) Axial view of the right distal femur showed the infiltration of the tumor to the anterior compartment of the muscles and displacement of femoral and popliteal vessels and nerves. (f) Necrotic area is also visualized within pathological fracture segment, seen on coronal image (white arrow).

**Figure 4.**
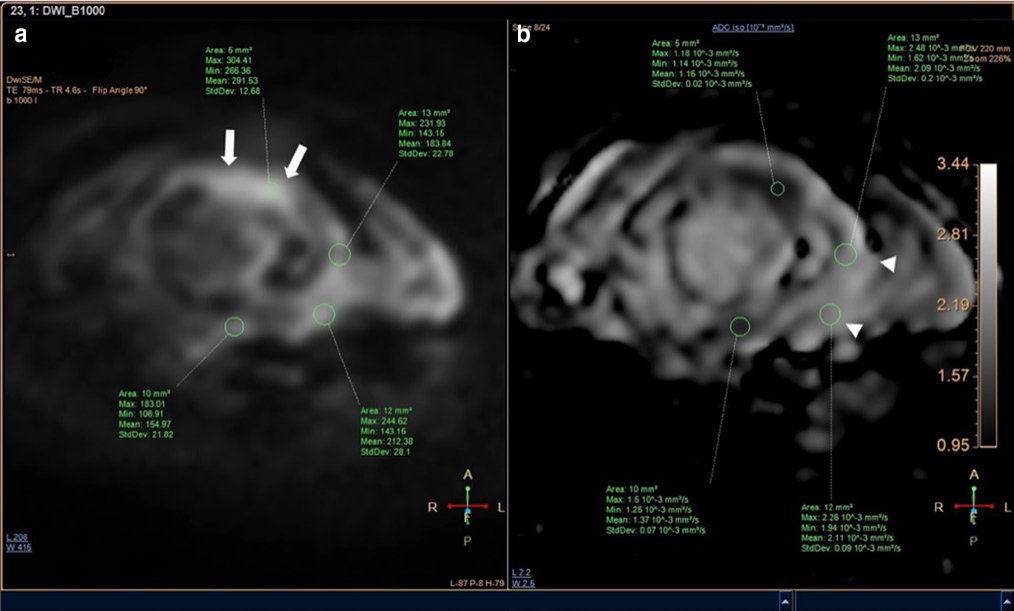
Initial MRI examination with DWI (a) and ADC mapping (b) MRI of the primary tumor showed remarkable restricted diffusion in the solid area (white arrows), with lower ADC values = 1.15–1.2 × 10^–3^ mm^2^/s in the solid area and higher ADC values 2.05–2.54 × 10^–3^ mm^2^/s in the necrotic area (white arrow heads). ADC, apparent diffusion coefficient; DWI, diffusion-weighted imaging.

**Figure 5.**
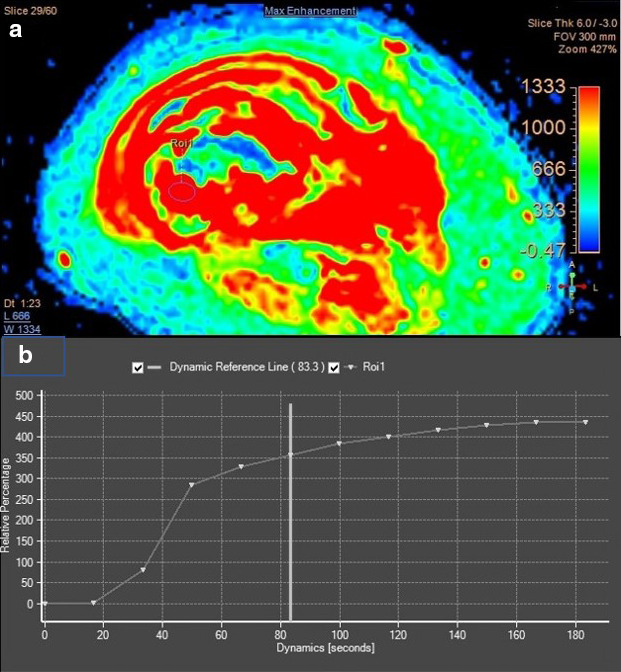
Perfusion MR imaging of initial MR examination showed early enhancement with mixed progressive enhancement and plateau pattern of enhancement (a, b)

**Figure 6.**
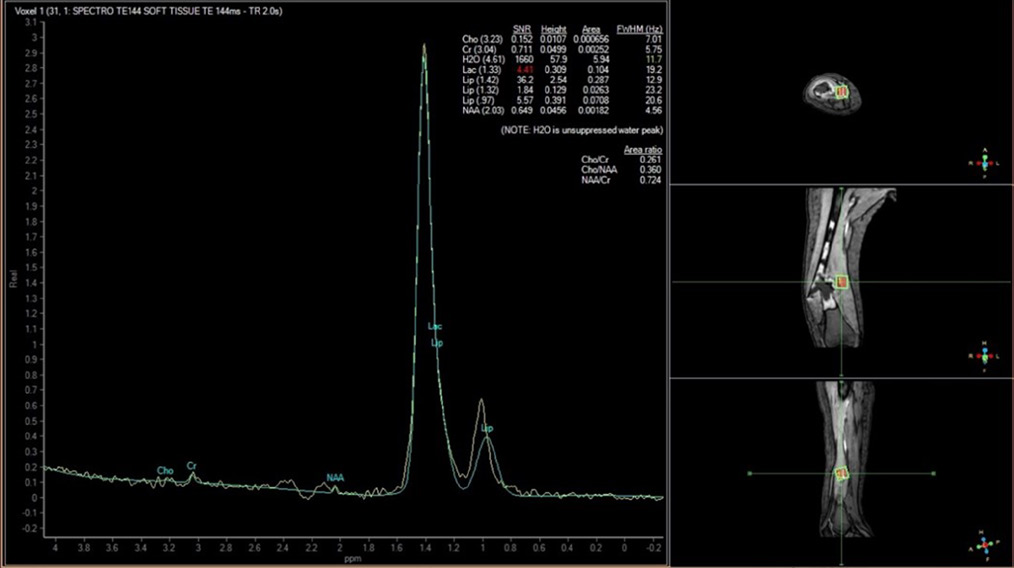
MR spectroscopy of initial MR examination showed significant elevation of lipid lactate and unremarkable elevation of choline metabolite.

MR imaging of the femur was then performed 3 weeks after second radiograph examination, with axial, sagittal, and coronal proton density fat saturation (PD FS) images; coronal T1 fast spin echo (FSE) and axial T2 fast relaxation fast spin echo (FRFSE) images; axial diffusion-weighted imaging (DWI) with apparent diffusion coefficient (ADC) followed by MR perfusion and MR spectroscopy; as well as post-Gadolinium contrast-enhanced 3D T1FS images. Bone destruction was found in one-third distal to the mid-femoral diaphysis with bulky soft tissue mass, about 6.044 × 3.662 × 13.254 cm, and pathologic fracture in distal femoral shaft. Intramedullary involvement was found to the mid-femoral shaft. Multiple skip lesions along fifth lumbar vertebra, right femoral shaft, left ischium bone, and left proximal femur were also detected. In the post-contrast images showed heterogeneous enhancement of the lesion with central necrotic area. The mass is shown to infiltrate the anterior compartment of the vastus muscles and displace the femoral and popliteal vessels and nerves to the posterior (Figure 3A–F). DWI showed restricted diffusion area with ADC mapping of 1.15–1.2 × 10^–3^ mm^2^/s in the solid area, which suggested a malignancy, and 2.05–2.54 × 10^–3^ mm^2^/s in the necrotic area (Figure 4). MR perfusion showed initial enhancement with mixed progressive enhancement and plateau pattern of enhancement (Figure 5). There were unremarkable elevation of choline metabolite and significant elevation of lipid lactate in the necrotic area in MR spectroscopy (Figure 6). From MRI, findings has indicated two differential radiological diagnoses of synchronous multifocal osteosarcoma and primary bone lymphoma.

**Figure 7.**
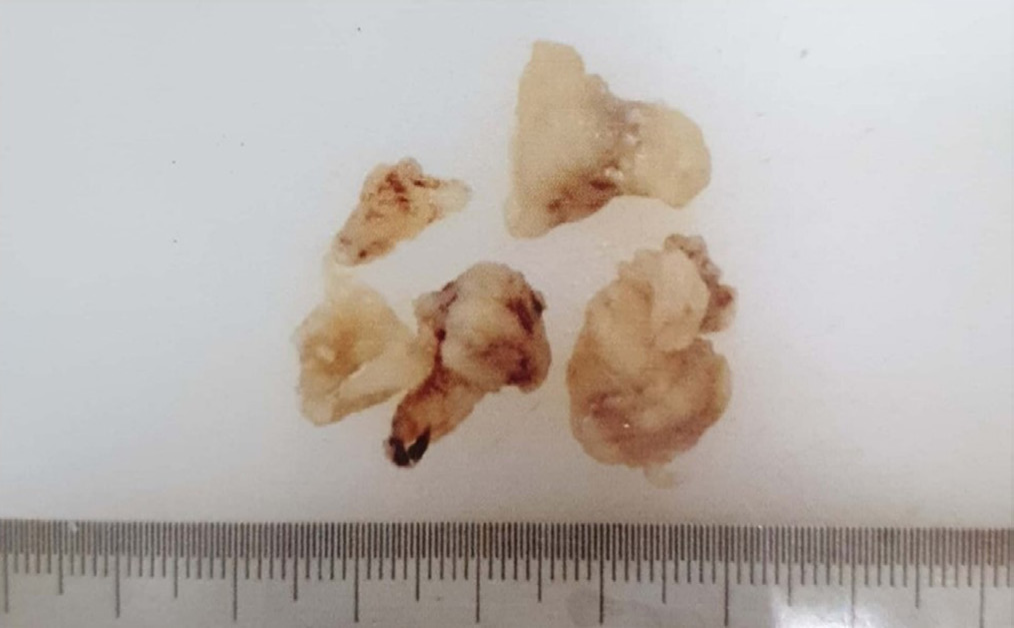
Macroscopic specimens obtained during open biopsy showed several irregular greyish tissues of bone tumor.

**Figure 8.**
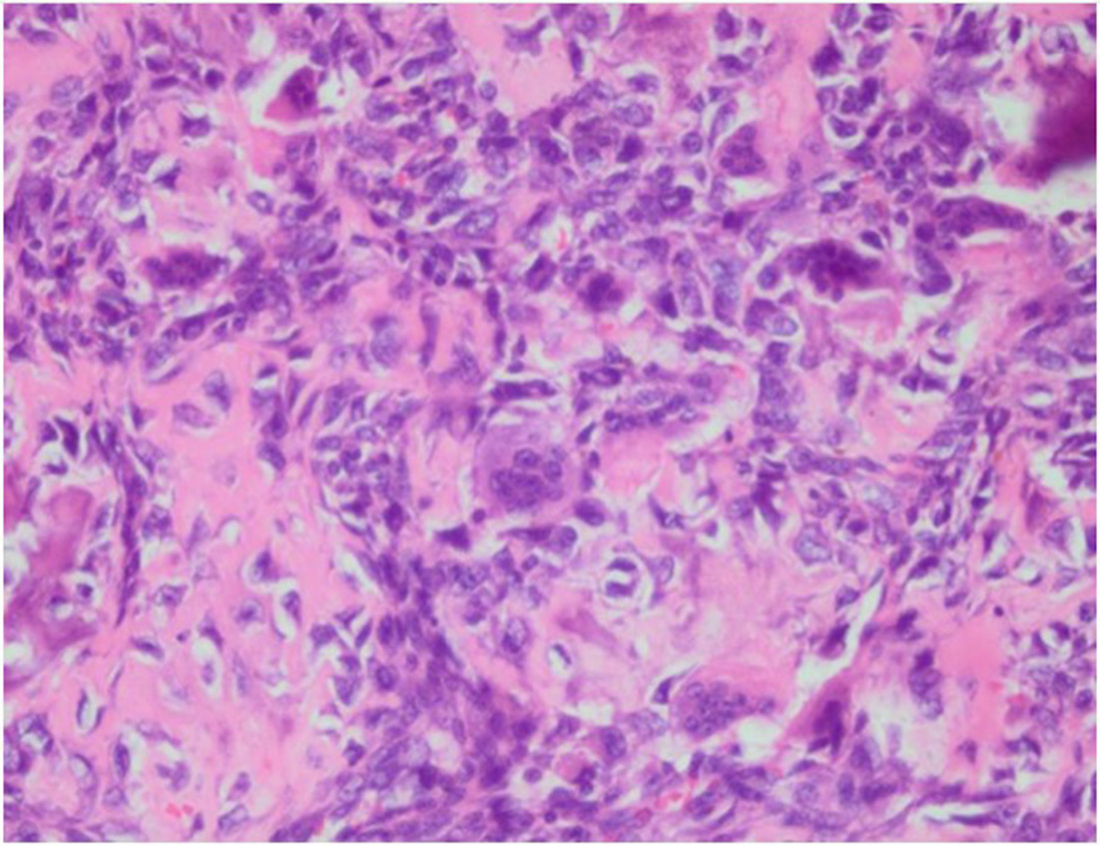
Microscopic examination of the specimen demonstrated anaplastic cells proliferation, arranged diffusely in the osteoid matrix forming lace-like appearance.

After having several imaging procedures, then the open biopsy had been done and the specimen obtained during biopsy was several irregular grayish tissues sized 0.5–1.5 × 1.5×1 cm (**Figure 7**). Microscopic examination showed anaplastic cells proliferation with round oval to pleomorphic spindle, hyperchromatic nucleus. The cells were arranged diffusely in the osteoid matrix, forming lace-like appearance (**Figure 8**). This feature supported the diagnosis of synchronous multifocal osteosarcoma.

Then, patient had been underwent two series chemotherapy using methotrexate, doxorubicin, and cisplatin, with mild side-effect of nausea, loss of appetite and mouth sores. No significant impact to her blood count or serious complication was occurred.

**Figure 9.**
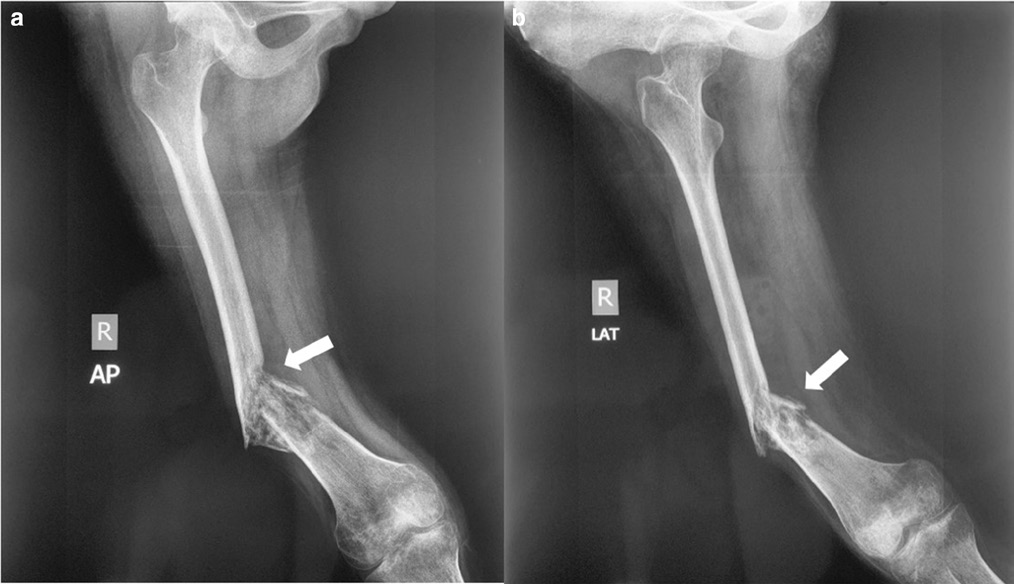
Follow-up radiograph (10/03/2020) of the destructed part of the femur demonstrated good response after chemotherapy with partial union fracture with callus formation and reduction of the mass size of the right thigh (white arrows). (a) Anteroposterior image and (b) lateral image.

Patient felt reducing pain on the her distal thigh 4 months later after following two series of chemotherapy, then routine follow-up radiograph of the right femur has been obtained, showed partial union of the destructed part with the formation of callus and reduction of the mass size (Figure 9) . The patient was doing well during subsequent visits.

**Figure 10.**
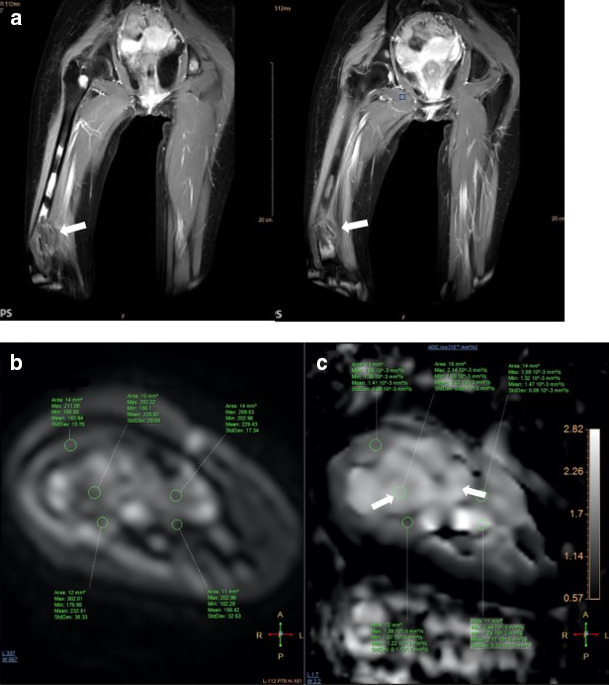
Follow-up MRI evaluation (26/03/2020) with T1 Fat Sat MRI images with intravenous gadolinium contrast (a) dan DWI (b) as well as ADC mapping (c) . On T1fat sat post-contrast showed reduction of the overall mass size and the composition of the viable area (white arrows) (a) Recent DWI with ADC mapping MRI demonstrated a value of 1.22–1.41 × 10^–3^ mm^2^/s in the solid area and a value of 2.02–2.17 × 10^–3^ mm^2^/s in the necrotic area (white arrows) (b, c). ADC, apparent diffusion coefficient; DWI, diffusion-weighted imaging.

**Figure 11.**
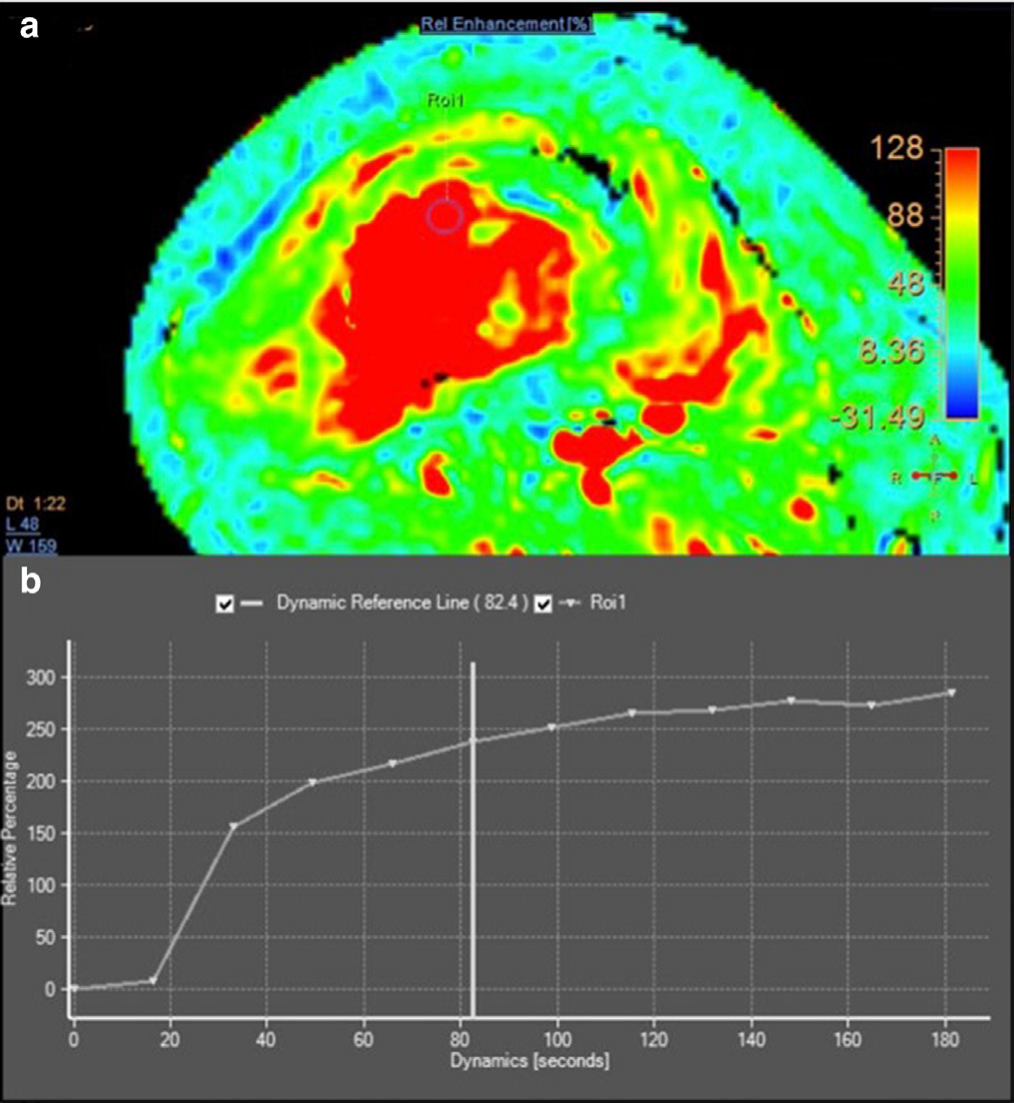
Follow-up perfusion-weighted MR imaging showed decreased the rate of enhancement compared to previous MRI with gradual enhancement TIC pattern.

**Figure 12.**
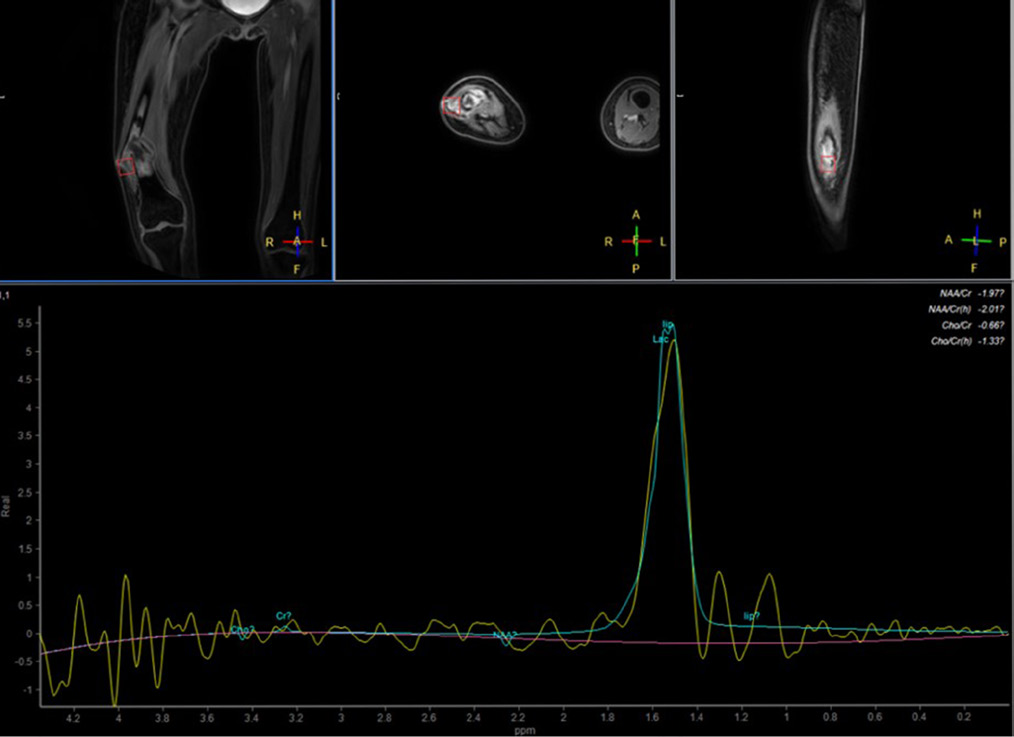
Follow-up MR spectroscopy showed non-significant increase of choline and significant elevation of lipid lactate compared to the previous MRI.

Follow-up advanced MRI had also been obtained 2 weeks after the latest radiographs, demonstrating reduction of the overall mass size and the composition of the viable area (Figure 10). DWI demonstrated ADC mapping of 1.22–1.41 × 10^–3^ mm^2^/s in the solid area and 2.02–2.17 × 10^–3^ mm^2^/s in the necrotic area (Figure 10). MR perfusion-weighted imaging showed decreased rate of enhancement compared to the previous MRI (Figure 11). MR spectroscopy showed no significant elevation of the choline metabolite and significant elevation of lipid lactate composition compared to the previous result (Figure 12).

## Discussion

Multifocal osteosarcoma is a rare case in which two or more tumors occur at multiple points without visceral metastasis. There are two types of multifocal osteosarcoma, synchronous (more than one lesions at presentation, usually develop within 6 months) and metachronous (new tumor developing after the initial treatment, usually over 6 months interval).^7^ The incidence of synchronous multifocal osteosarcoma is around 1–3%..^4,7^

Several conditions are required to establish the origin of multifocal osteosarcoma: absence of previous systemic bone pathology, negative history of radiation exposure, simultaneous appearance of the lesions in the affected bones, and absence of pulmonary metastasis.^8^ In our case, unremarkable history of the patient and family supported one of multifocal osteosarcoma criteria.

In 1969, Amstutz HC^6^ described this multifocal osteosarcoma as the presence of one primary tumor and several skeletal lesions. He describd a synchronous form (Type I child/adolescent high grade tumor and Type II adult low grade tumor) and a metachronous form (Type III, subdivided into IIIa and b—early and late presentation of the second lesion).^6^

In patients with synchronous multifocal osteosarcoma, the primary tumor has features suggestive of a primary osteosarcoma, usually presented as being more extensive than the other lesions. The smaller lesions, instead, mimic skeletal metastases on X-ray and MRI, presented as purely sclerotic or heavily mineralized metaphyseal lesion, with narrow transition zone, no cortical destruction or soft tissue mass, nor malignant periosteal reaction, in contrast to the metachronous group, which most of the lesions appeared similar to the presentation of primary osteosarcoma.^9–11^

10 years later, Mahoney proposed an other classification of mutilple osteosarcoma in four groups (A–D) based on age, histology, and time of presentation of lesions. Group A included childhood and adolescent patients with multiple synchronous tumors; Group B comprised adults with low grade multiple simultaneous lesions. Groups C and D consisted of metachronous lesions with a time interval between the first and the second lesion of less than 24 months (Group C) and greater than 24 months (Group D).^12^

In the presented case, multiple lesions appeared at the time of diagnosis, with one primary tumor (right distal femur) which led to the presentation, and synchronous lesions in the several points surrounding the primary tumor with well-defined border and without tumor extension. The primary tumor was first shown in the plain radiograph in osteolytic-dominant pattern rather than the usual sunburst appearance, although the Codman triangle could be found.^13^ However, the synchronous lesions were detected after the first MR imaging was performed, instead of the plain radiograph, as mentioned by Yang,^14^ that skip metastases in many cases were possibly missed due to lacking of imaging modalities. Compared to the first MR images, in which the multiple lesions were already seen, the later MR images revealed the primary tumor being more extensive with pathological fracture and additional skip lesions in the pelvic bone.

Synchronous type principally involves long bones in symmetric distribution,^15^ while the metachronous group prefers the axial skeleton, such as pelvis, spine, shoulder girdle, skull, synchronous type usually occurs in typical osteosarcoma location, such as distal femur, proximal tibia, or humerus.^16^ In the presented case, the main tumor was precisely located in the usual synchronous type location, however, multiple skip lesions could also be seen in the pelvic bone.

Zhang reported a rare case of synchronous multifocal osteosarcoma which occurred in the maxilla and the mandible.^16^ Furthermore, there were three similar cases involving axial bones in 15, 16, and 17-year-old girls, respectively. The first case was a synchronous multifocal osteosarcoma occurred in the right ilium and left ramus of mandible.^17^ The second case were involving right ramus of mandible and left mid-shaft of the femur.^18^ The last case was a multiple metastatic osteosarcomas involving vertebrae, right zygomatic bone, right clavicle, sternum, right and left humerus, right tibia, right and left lungs, meninges, and mandible, with primary tumor in the left fibula.^19^

Most recent reviews concluded the multifocal osteosarcoma being a metastatic process rather than multicentric origin.^20–22^ One of the reason was the presence of one dominant tumor that led to presentation, which could be considered as a primary tumor.^4^ Enneking and Kagan^23^ described that the tumor cells may invade the marrow sinusoids and embolize both proximal and distal intraosseously, whereas the route for transarticular skips was less defined. They suggested the mechanism might be mimicking the vertebral spread of the prostatic tumor via Batson venous plexus. Alternatively, Hatori et al have demonstrated lymphatic spread to the lungs, giving another possible route.^24^

Furthermore, the response of both primary and synchronous/metachronous lesions to chemotherapy was similar, and this evidence supports the bone-to-bone metastases theory.^24–31^

The main limitation in the presented case was the absence of thoracic CT, which is necessary in every osteosarcoma case. Although the routine chest radiograph was appeared to be normal, it was not as sensitive as CT scan in demonstrating small pulmonary lesion.^23^

In most settings, the imaging study for diagnosis and staging of osteosarcoma was mainly done using CT scan and MRI. These two modalities were superior for assessing the anatomic extent of the primary lesion. However, a radionuclide study is required for accurately assessing the metastatic disease and metabolic activity of the primary lesion. Whole body bone scintigraphy using 99m technetium-MDP has a high sensitivity in staging metastatic disease.^9,10^ The presence of multiple bone lesions without any lesion in lung fields confirms the diagnosis of multifocal osteosarcoma differentiating it from primary osteosarcoma with multiple metastases. Furthermore, pre-treatment MDP scan can help assess the effectiveness of the therapy when compared to the post-treatment scan.^9,10^ This modality can be used for this purpose for further similar cases.

Single lesion osteosarcoma and multifocal osteosarcoma have different disease courses and survival times, so it is important to make a differential diagnosis. Prior chemotherapy era, synchronous multifocal osteosarcoma was considered fatal within few months. Although in ordinary osteosarcoma without detectable metastases, surgery combined with chemotherapy significantly improves the success of treatment to 60–70%, the prognosis for synchronous multifocal osteosarcoma remains extremely poor in spite of the combined surgery and chemotherapy.^24–31^ However, in our case, good response had been established following chemotherapy. This might be proven in the second MR image which showed the large necrotic area in the center of the mass, furthermore, MR spectroscopy showed remarkable elevation of lipid lactate composition which indicated the necrotic area being extensive, without significant elevation of choline metabolite. Furthermore, after series of chemotherapy, latest follow-up plain radiograph showed union of the destructed bone with formation of callus and reduction of the mass. Advanced MRI obtained afterward demonstrated the reduction of viable tissue composition.

## Conclusion

The case of synchronous multifocal osteosarcoma in an Indonesian girl has been reported. This diagnosis was based on MRI finding, which showed multiple lesions and the primary tumor, and proven by the biopsy which supported osteosarcoma. Although many literatures stated the prognosis of synchronous multifocal osteosarcoma being poor, favorable response has been achieved following chemotherapy. The latest follow-up imaging showed reduction of the overall mass size and the cell viable composition.

## Learning points

- Synchronous multifocal osteosarcoma is a rare condition, with a reported incidence of 1–3%.
- Imaging evaluation using MRI is essential to make diagnosis and to evaluate the efficacy of chemotherapy.
- Advance MRI using DWI, ADC, MR perfusion and MR spectroscopy can give additional diagnostic information, providing a non-invasive assessment of tumor viability. Therefore, it may help monitor treatment response.
- Post-treatment changes cause decreased vascular permeability of the tumor vessels as well as viable tumor cell presentation.
- Synchronous multifocal osteosarcoma prognosis remains extremely poor in spite of the combined surgery and chemotherapy. In this case, there was a good response.
